# COVID-19 as WATER? The functions of WATER metaphors in the metaphorical representation of COVID-19

**DOI:** 10.1371/journal.pone.0292806

**Published:** 2023-11-09

**Authors:** Zhibin Peng, Yating Yu, Dennis Tay

**Affiliations:** 1 Foreign Language Research Department, Beijing Foreign Studies University, Beijing, China; 2 The Department of Communication, Faculty of Social Sciences, University of Macau, Macau SAR, China; 3 Department of English and Communication, The Hong Kong Polytechnic University, Hong Kong SAR, China; Kitami Institute of Technology, JAPAN

## Abstract

Previous studies have examined WATER metaphors in different discourses, yet there has been limited focus on understanding the functions of these metaphors in constructing discourses. To address this research gap, the present paper utilised the metaphor identification procedure developed by the Pragglejaz Group and the Weak Differentiating Model to investigate WATER metaphors in the Coronavirus Corpus. The study reveals that WATER metaphors can be used flexibly for multiple purposes. These include explaining the many pandemic stages, describing the occurrence and performance of the pandemic, explaining how COVID-19 affects human life, rallying the public to take precautions against contagion, and communicating risk. This research highlights the diverse functions that WATER metaphors served in conveying intricate information and influencing public perceptions throughout the COVID-19 pandemic.

## Introduction

Metaphors serve as valuable tools for discussing and explaining information in relation to the COVID-19 pandemic. Since its emergence in December 2019, a range of metaphors, including CHESS [1], DIRECTION [2], FIRE [3], FRIEND/COMPANION [4], MONSTER [5], MOVEMENT [6], SUPERHERO [7], WAR [8–13], and others, have been employed in public discourse surrounding this health crisis. Linguistic metaphors refer to specific instances of metaphorical phrases found in language, whereas conceptual metaphors refer to the underlying cognitive mechanisms that influence our comprehension of abstract or difficult concepts by mapping them onto more tangible or familiar domains [28]. Linguistic metaphors are written in italics and conceptual metaphors are written in capitals. Individuals from diverse backgrounds, including healthcare professionals, journalists, political leaders, and the general public, use various figurative expressions to describe the pandemic [3, 14–20]. Scholars specialising in linguistics have examined the conceptual metaphors employed in different forms of COVID-19 discourse, with the WAR metaphor emerging as the most prevalent [8–13].

Whilst earlier research has proposed that WAR metaphors ought to be avoided in the discourse surrounding COVID-19 and substituted with more effective metaphors that emphasise empathy and collaboration [3, 13, 21], scholars worldwide have gathered alternative metaphors to the WAR metaphor [3, 20]. In this investigation, we present an alternative metaphor, namely the WATER metaphor, in order to examine its usage in the context of describing COVID-19. People possess familiarity with the fluidity of water, as well as the transactional characteristics of water and other natural elements. WATER metaphors can act as a means to discuss the intricate and ever-changing nature of the pandemic. Although certain prior studies have analysed WATER metaphors in various discourses [22–27], few have explored the functions of WATER metaphors. We have employed COVID-19 discourse as a case study to examine the functions of WATER metaphors in describing COVID-19. Our objective is to address the following research question: How are WATER metaphors utilised in COVID-19 discourse, and what functions do they serve?

## Literature review

This section will present a review of the literature concerning the theoretical concepts as well as metaphor studies of WATER metaphors and the COVID-19 discourse.

### The theoretical concepts

For centuries, metaphor has been regarded as a rhetorical embellishment that departs from everyday language. Moreover, metaphor was considered primarily as a matter of language rather than a question of cognition or behaviour. The conventional perception of metaphor was subsequently contested by the cognitive perspective on metaphor in the 20th century, particularly by the Conceptual Metaphor Theory (CMT).

CMT is a significant theoretical framework proposed by cognitive linguists Lakoff and Johnson [28] in their collaborative work *Metaphors We Live By*. In contrast to the traditional view that considers metaphor as a language phenomenon only, CMT emphasises the cognitive nature of metaphor. Lakoff and Johnson [28] discovered that metaphor exists “not just in language but in thought and action” (p.3). They also suggested that our ordinary conceptual system is “fundamentally metaphorical in nature” (p.3), and that “the essence of metaphor is understanding and experiencing one kind of thing in terms of another” (p.5).

CMT is a powerful theoretical tool widely employed by cognitive-linguistic researchers who have explored all kinds of discourses over the past few decades, including the COVID-19 discourse. We can discuss and consider one item in relation to another because these two items seem to have some parallels [29]. For instance, we refer to ILLNESS as WAR because we recognise the similarities between dealing with illness and waging WAR [30]. Despite the fact that the two notions come from distinct semantic domains, our mind recognises the structural connections between them. These connections are known as “cross-domain mappings” caused by metaphors. For example, when we say, “We are fighting against the illness” or “We will defeat the illness”, we are drawing a comparison between our battle against the illness and a violent conflict with an adversary, with the aim of minimising its impact. One of the fundamental concepts of CMT is that metaphorical language is a surface phenomenon that stems from conceptual thought structures. Figurative expressions such as the two sentences mentioned above are linguistic realisations of the conceptual metaphor–ILLNESS IS WAR. The WAR metaphor may not be a suitable metaphor for illnesses, as it conveys a negative and toxic attitude towards treatment [31].

The Strong Differentiating Model (SDM) and the Weak Differentiating Model (WDM) are types of Cognitive Models proposed by Wang [32] to explain the structuring of discourse from the perspective of Cognitive Linguistics, based on the basic presupposition held by cognitive linguists that Cognitive Models support the structuring of discourse. As key terms in Cognitive Linguistics, cognitive models are structured mental constructs that provide a way to describe the structured encyclopaedic knowledge that is intrinsically linked to linguistic information [33]. Wang [32] argues that since we are generally unaware of the structuring role of Cognitive Models on our behaviour, the vast majority of people do not realise the existence of the cognitive model of SDM, but through careful and in-depth observation, it is easy to see the deep imprint of SDM in social, economic, and cultural development. Discourse is not an impartial entity; rather, it possesses a constructive quality. Stibbe [34] draws a distinction between “destructive discourse” and “beneficial discourse” within the framework of ecological linguistics. Destructive discourses are those that stand in opposition to social equity and justice and undermine various facets of social interaction. They are widely seen as having a detrimental impact on the dynamics of social engagement. SDM has the capacity to shape the formation of discourses. The discourses that emerge from SDM are classified as destructive, as they actively contribute to the erosion of social interaction. The COVID-19 discourse framed within the WAR metaphor is grounded in SDM, which highlights the confrontation between humanity and the pandemic, thus drawing the public’s focus to the conflicts between the pandemic and humans [13].

Wang [22] argues that the construction of discourse is sometimes influenced by SDM, which might have socially destructive forces. We should base the construction of discourse on another cognitive model called WDM, which goes against SDM. According to Wang [32, 35], WDM is based on the Prototype Theory of Categorisation. Ideas in this direction were first formulated in philosophy by Wittgenstein [36] and Quine [37, 38], and then in psychology by Rosch [39] and others. According to the Prototype Theory of Categorisation, Wittgenstein [36] demonstrated that a concept such as a “game” could not be adequately defined by a set of sufficient and necessary conditions. Instead, the members of such categories are related by what he called family resemblance. Games are a classification encompassing entities that share common characteristics, rendering them akin to one another. Cards, balls, running, and the like are all considered games as they exhibit overlapping traits and elements. The demarcation lines between the constituents of a given category are not well-defined, but rather possess a degree of ambiguity. For instance, the Cognitive Model underlying sharing pedagogics [40] and sharing economy [41] is WDM, and the discourse based on it has socially beneficial effects [35]. Wang [35] suggests that structuring discourses exclusively based on SDM may have potential drawbacks for society, while structuring discourses based on WDM may have potential advantages. Given this fundamental theoretical assumption, we propose that the WATER metaphor, rooted in WDM, could serve as a viable alternative to those based on SDM. We have chosen this model as the theoretical foundation because it highlights the reasons why the WATER metaphor could be a suitable option when constructing pandemic discourse. It is crucial to consider if the WATER metaphor is appropriate in areas that frequently experience floods or tsunamis because these calamities can cause major destruction and human casualties. A separate study that considers the unique cultural, socioeconomic, and contextual aspects of these places is suggested.

### Metaphor studies of WATER metaphors and the COVID-19 discourse

Some previous studies have examined WATER metaphors in various discourses. Most studies describe that WATER metaphors were used negatively in daily lives to convey certain ideologies. For instance, Mujagić [24] used the Metaphor Identification Procedure VU University Amsterdam (MIPVU) approach [42] to investigate WATER metaphors describing migrants, migration, and countries that receive migrants in news articles about the Refugee crisis. She finds that the metaphor MIGRATION AS DANGEROUS WATERS is frequently used to promote discriminatory viewpoints of migrants in Bosnian-Herzegovinian, British, and American news media. Similarly, Taylor [27] conducted a case study on the metaphor of MIGRANTS ARE WATER in the British *Times* newspaper from 1800 to 2018. She finds that the WATER metaphor has been extensively used in the debate regarding how migration is portrayed in the UK press, but she also shows that opinions can vary greatly over time.

A few studies demonstrate that WATER metaphors can also be used positively. One example is Fox’s [23]. She engaged in a theoretical discussion of the concept of occupation in relation to the use of metaphors. She argues that the use of the WATER metaphor can help bridge concepts together and expand knowledge, and that this metaphor can help us understand the fundamental properties of occupation. Although these studies have explored WATER metaphors in different discourses, few have specifically investigated the functions of WATER metaphors in constructing discourses. To address this research gap, we used the COVID-19 discourse as a case study to examine the functions of WATER metaphors in constructing the discourse.

Following the outbreak of the COVID-19 pandemic at the end of 2019, linguists have begun to take notice of the language used to express the pandemic. CMT has also been utilised by many researchers to comprehend the COVID-19 discourse. They have revealed the types of conceptual metaphors employed in framing the pandemic. In previous studies, the COVID-19 pandemic has been framed as CHESS [1], DIRECTION [2], FIRE [3], FRIEND/COMPANION [4], MONSTER [5], MOVEMENT [6], SUPERHERO [7], WAR [8–13], and so forth. While the conceptual metaphors used in framing COVID-19 are varied, previous studies almost unanimously argue that WAR metaphors are ubiquitous in the COVID-19 discourse and are the most widely used. Given that COVID-19 is a new, urgent, and severe global issue, fighting, battling, and war metaphors have been used to discuss the epidemic, regardless of the language used [43–45].

Most previous research has suggested avoiding WAR metaphors but has not provided guidance on what metaphors should be used for discussing COVID-19. Addressing this gap, two Spanish academics, Paula Pérez-Sobrino and Inés Olza, initiated The #ReframeCovid campaign on Twitter, later joined by Veronika Koller and Elena Semino at Lancaster University. The purpose of this campaign was to gather alternative metaphors to replace WAR metaphors [20]. So far, the initiative has collected a variety of metaphors in different languages. However, these examples remain tentative and scattered, with contributors worldwide seeking further clarification on the appropriateness of these substitute metaphors.

Additionally, Semino [3] suggests that FIRE metaphors are particularly suitable and valuable for conveying information about the pandemic. She argues that fires are dynamic and come in various forms, such as house fires, dumpster fires, and forest fires, involving multiple components and participants, including arsonists, forests, firemen, casualties, and more, and having a distinct progression. Furthermore, employing both quantitative and qualitative methods, Busso and Tordini’s [19] study compared the themes and metaphors utilised by journalists during the initial and subsequent phases of the coverage of the Italian Government’s response to the pandemic in Italian online newspapers. They noted a notable change in the covered themes between Phases 1 and 2, along with intriguing similarities in the metaphors employed to address each subject. Despite the valuable insights of these studies, they did not delve into the functions of metaphors, such as the one we suggest (“COVID-19 as WATER”).

Wang and Zheng [13] suggest that the discourse surrounding COVID-19 based on the PANDEMIC IS WAR metaphor can be labelled as “hardcore” discourse, which emphasises the conflict between humans and the pandemic, leading to an incorrect perception of the pandemic, the virus, and their relationship with humans. Wang [35] contends that structuring discourses based on SDM can be detrimental to society. The primary reason why the WAR metaphor has adverse effects on communication about the pandemic is that it relies on SDM. Therefore, it may be beneficial to explore alternative metaphors such as the WATER metaphor in addition to FIRE metaphors suggested by Semino [3] for communicating about the pandemic, which is currently being researched.

### Data and method

To examine the usage of WATER metaphors in COVID-19 discourse, this study analyses WATER-related lexical units in the Coronavirus Corpus (CC; https://www.english-corpora.org/corona/), which is a compilation of texts from diverse sources worldwide, spanning from January 2020 to December 2022. The corpus, initially released in May 2020, contained around 13.3 billion words as of the data collection cut-off date (16th January 2022), and it continues to grow by three to four million words daily.

To generate a list of potential WATER-related metaphors, we referred to Lakoff et al.’s [46] Master Metaphor List and Taylor and Kidgell’s [47] metaphor list on framing pandemics. The resulting list consists of 31 metaphors and should be regarded as tentative in nature rather than a comprehensive compilation. Subsequently, we analysed each metaphor in the CC and established the final list for this study as follows:

*afloat*, *boat*, *bubble*, *canoe*, *dive*, *drift*, *drown*, *ebb*, *float*, *flow*, *immerse*, *life jacket*, *paddle*, *plunge*, *pool*, *raft*, *reservoir*, *rise*, *sink*, *smooth*, *stormy*, *subside*, *surface*, *surge*, *swim*, *tide*, *undercurrent*, *wake*, *wave*.

Next, we utilised the final list of WATER-related metaphors to search for concordances and then manually analysed them. Considering that gathering all concordances would yield millions of examples, potentially affecting the precision of our analysis, we restricted ourselves to the top 200 concordances for each metaphor, thus forming a more manageable dataset. Initially, the first two authors employed the Pragglejaz Group’s [48] proposed metaphor identification procedure (MIP) to identify the metaphorical usage of WATER-related terms, including WATER-related similes and other “direct” metaphors, but not those that applied to issues outside of COVID-19 [49]. We then discussed the potential variables for the functions of all the identified metaphors and separately analysed their context-specific functions. For data analysis, we used QSR International’s NVivo 12 Pro software, which enables data annotation and generates the inter-rater reliability rate. The Kappa Coefficient test yielded a result of 0.88, indicating a high level of agreement between the two authors [50]. Finally, we discussed and resolved any problematic cases to finalise our analysis.

## Results

The study identifies five main functions for which WATER metaphors can be employed flexibly (see Table 1), with Table 1 displaying the frequencies of these functions across the entire dataset. In this section, we will discuss how these functions are realised by utilising various WATER metaphors drawn from our corpus data.

**Table 1:** The five functions and their frequencies across the data.

Functions	Freq. (%)
different stages of the pandemic	283 (32.3)
the occurrence and performance of the pandemic	272 (31.1)
the impact of COVID-19 on life	175 (20.0)
measures for reducing contagion	116 (13.2)
conveying danger	30 (3.4)
**Total**	876 (100)

### Different stages of the pandemic

WATER metaphors in COVID-19 discourse are most frequently used to differentiate between different stages of the pandemic (32.3%). Of the 29 WATER-related terms, nine (i.e., *wave* 102, *subside* 79, *tide* 51, *ebb* 42, *pool* 3, *plunge* 1, *raft* 1, *sink* 1, *stormy* 3) serve this function, with *wave*, *subside*, *tide*, and *ebb* being the most prominent. *Wave*, modified by ordinal numerals such as first, second, third, fourth, or by next, another, etc., is the most used term to describe various stages of the COVID-19 pandemic (Examples 1).

The pandemic or virus is metaphorically likened to tides. Tides can cause extensive and irreparable damage due to their rapid growth and spread. Tides may *subside* or *ebb*, and the life cycle of tides can be used figuratively to distinguish between different pandemic severity stages, as measured by the number of new infections and the effectiveness (or lack thereof) of efforts to reduce those numbers. The conceptual metaphor is THE DECREASE OF PANDEMIC IS THE SUBSIDING/EBBING OF THE TIDE, as demonstrated in Examples 2, 3 and 4.

### Examples

1. The CNN anchor finally mentioned the controversy to his brother after ignoring it during at least 10 on-air interviews, but the governor quickly pointed to how there were nursing home deaths “all across the country” and said “we have to figure out how to do it better the next time” before the next virus ***wave*** occurs. (CC)
2. “The good news is here in Michigan, COVID-19 is starting to ***subside*** and the governor and Legislature are focused on how to get the economy going again”, he said. (CC)
3. They say that “it’s only when the ***tide*** cgoes out that you see who’s been swimming naked”, and in all manner of things COVID19 has certainly seen the ***tide*** go out. (CC)
4. And when so many people have lost so much, there is some guilt in adding a human to our lives. As the pandemic’s tide continues to ***ebb***, flow and evolve, we hope the exit of this portal is within sight. (CC)

### The occurrence and performance of the pandemic

The COVID-19 pandemic’s occurrence and performance are conveyed through 13 WATER-related words (31.1%), including *surge* 120, *rise* 74, *bubble 35*, *flow* 9, *drift* 7, *plunge* 7, *float* 6, *immerse* 5, *reservoir 5*, *surface* 5, *wave* 4, *undercurrent* 2, *tide* 1, with *surge*, *rise*, and *bubble* being the most prevalent. The term *surge*, denoting waves moving forward in motion, is widely used in public communication to characterise the outbreak of the COVID-19 pandemic. In Example 5, we observe the conceptual metaphor THE OCCURRENCE OF THE PANDEMIC IS THE SURGE OF WATER, while in Example 6, the rapid increase of infected cases is also metaphorically described as a *surge* of WATER with the conceptual metaphor THE INCREASE OF THE PANDEMIC CASES IS THE SURGE OF WATER (Example 6). The term *rise* is the second most frequently used term to convey this function. It is employed to describe the deterioration of the pandemic (Example 7). The WATER frame is employed in the corpus we analysed to depict both the widespread eruption of the pandemic and the emergence of sporadic cases of infection. For instance, when the pandemic is effectively managed, the occurrence of isolated infected cases is metaphorically likened to the *bubbling up* of WATER, as illustrated in Example 8.

5. President Joe Biden has unveiled a new “action plan” to confront the COVID-19 ***surge*** driven by the spread of the delta variant. It mandates vaccines for federal workers, and contractors and certain healthcare workers require employees at companies with 100 or more workers to be vaccinated or tested weekly. (CC)
6. With students heading back to campus, some universities are seeing a ***surge*** in covid cases. It was unfortunate when we came here and covid started exploding. (CC)
7. The ***rise*** of Covid-19 will lead to unprecedented rates of absentee voting in the 2020 election. (CC)
8. The return of college football brought up some mixed emotions for America. We had massive crowds and the atmospheres were electric, but they also stir up some natural anxiety. There have been significant outbreaks ***bubbling up*** across the country, especially in schools. (CC)

### The impact of COVID-19 on life

Describing the impact of COVID-19 on human life represents the third most common function for the WATER metaphors, accounting for 20.0% of occurrences. In our list, we have identified 18 words related to water that serve this particular function: *wake* 67, *afloat* 30, *plunge* 21, *subside* 14, *dive* 9, *drown* 5, *undercurrent* 5, *drift* 4, *flow* 4, *ebb* 3, *reservoir* 3, *sink* 3, *surface* 3, *surge* 3, *immerse* 2, *raft* 2, *smooth* 2, and *boat* 1. Among them, *wake* is the most frequently employed, often used in the phrase *in the wake of* the pandemic or COVID-19, which is a dead metaphor. After the pandemic outbreak, the entire country is facing an unprecedented situation. COVID-19 has affected almost all aspects of life, such as education (Example 9), business (Example 10), and economy (Example 11). The term *afloat* is used to describe how the pandemic has affected economic life. Faced with the threat of COVID-19, businesses are striving to stay *afloat* (Example 10). Additionally, *plunge* is mainly used to describe the impact of COVID-19 on economic life, such as recession (Example 11). *Subside*, on the other hand, is utilised to describe the reduction of the impact of COVID-19 on life (Example 12).

9. Addressing the ceremony, he said the country is facing unprecedented situation in the ***wake*** of coronavirus and this initiative will help children get education who are staying at home due to closure of schools in the ***wake*** of coronavirus. (CC)
10. She has weathered the organization’s near demise, worked through the Great Recession and lately been trying to devise ways to help businesses stay ***afloat*** during the coronavirus pandemic. (CC)
11. With markets on the edge, signs have started to mount that governments around the world are awaking to the need for stimulus measures to combat the virus, which is threatening to ***plunge*** the global economy into recession. (CC)
12. This optimism is likely driven in part by the 70 percent of executives who believe the negative impact of COVID-19 will ***subside*** during 2021. (CC)

### Measures for reducing contagion

Preventing the spread of the pandemic and reducing contagion is a key and necessary topic (13.2%) for the global flu-like pandemic. This function is achieved through 19 words: *raft* 31, *canoe* 19, *smooth* 9, *tide* 9, *bubble* 8, *pool* 6, *surge* 6, *life jacket* 5, *float* 4, *flow* 4, *drown* 3, *boat* 3, *subside* 3, *dive* 2, *drift* 2, *immerse* 2, *sink* 2, *plunge* 1, and *surface* 1, with *raft* and *canoe* being the most prominent. The term *raft* is mainly used in the phrase *a raft of measures* to discuss all kinds of measures to control the pandemic (Example 13). *A raft of* is already an established dead metaphor that finds usage not only within the WATER frame but also across other frames. *Raft* is also metaphorically employed in the COVID-19 discourse to refer to the quarantine resulting from the pandemic, as exemplified in Example 14. In Example 15, *life jacket* is employed in the pandemic discourse to metaphorically allude to medicine that holds the potential to treat COVID-19 disease. To call for “restraint and self-control”, people are thought to be “in the same *canoe*”. Therefore, people are advised to unite to do the right things to avoid sinking the *canoe* (Example 16).

13. The duo received the jabs immediately after the President had addressed the nation and introduced a ***raft*** of measures to contain the spread of the virus. (CC)
14. We could be our pod, be devoid of contact with others, and cling together as if on a ***life raft*** as the virus swept across the nation. (CC)
15. Since then, molnupiravir has been viewed as the newest ***life jacket*** in a pandemic that has left people in the U.S. exhausted by its longevity and the complicated politics, economics, and social challenges of living in this new normal.
16. It calls for restrain and self-control. We should turn these messages into a positive spin and encourage one another to do the right things. We are in the same ***canoe***. If the ***canoe*** sinks, we will all swim. We pray that no one will ***drown***. (CC)

### Conveying danger

In our data, this function (3.4%) is conveyed by nine words/phrases: *life jacket* 8, *reservoir* 7, *surge* 7, *drown* 2, *sink* 2, *paddle 1*, *plunge* 1, *swim* 1, and *tide* 1, with *life jacket* and *reservoir* being the most prominent. In the WATER frame, the pandemic is metaphorically conceptualised as the *tide* or *wave*, as illustrated in the first function. Human beings facing a surging *tide*, as found in the corpus, may be *drowned* in it. People nearby have *drowned* as a result of the WATER’s (especially *tides*) swift spread, difficulty in control, and large size. These qualities can be used as metaphors to illustrate the potential harm of COVID-19. For instance, the threat of the COVID-19 pandemic is expressed by the phrase “the *waves* of death and destruction”, which is threatening to *drown* humanity (Example 17). To promote the COVID-19 vaccination campaign, those who have been vaccinated are likened to having *life jackets*, while those who are unvaccinated do not have *life jackets* and are therefore at risk of drowning (Example 18).

Following the release of various vaccines, governments worldwide are advising people to get vaccinated to achieve herd immunity. When the majority of the public has been vaccinated, those who refuse to be vaccinated are metaphorically described as *a reservoir* of infection, as they pose a threat to others (Example 19). The metaphorical use of *sink* in Example 20 illustrates the impending risks presented by new COVID-19 variants.

17. We do not know why after over 13 crores recorded Covid-19 infections, 30 lakh deaths, nearly 700 million vaccine doses given worldwide, including in India, the ***waves*** of death and destruction keep coming, crashing on the rocks of fear and speculation, threatening to ***drown*** humanity in its worst nightmare. (CC)
18. Knowing the decision was coming, Mike Ryan, executive director of the World Health Organization’s Health Emergencies Programme, described it in a press conference a few hours in advance like this: “We’re planning to hand out extra ***life jackets*** to people who already have ***life jackets*,** while we’re leaving other people to ***drown*”.** Globally, more than 5 billion people remain unvaccinated. (CC)
19. Prof Read doubts vaccinating children on a mass scale will significantly decrease transmission of COVID-19 to adults. “Whereas with certain infectious diseases, children are a ***reservoir*** of infection, and they transmit the infection to adults, in this case for some curious reason it appears to be the other way around”, he said. (CC)
20. For example, business confidence “was on a tear in May and June,” Colyar said, but the renewed rise in infections has put that momentum in jeopardy. While “no one seems to think sweeping restrictions are coming back,” he continued, the risks posed by new Covid variants are starting to ***sink*** in.

## Discussion

The previous section analysed the use of WATER metaphors in COVID-19 discourse by examining their usage. The cognitive model behind the PANDEMIC IS WATER metaphor is WDM. The virus is to people what WATER is to humans. This section will discuss, from a macro perspective, the interrelationship between the WATER metaphor and the WDM cognitive model and its suitability for pandemic communication.

While the examples discussed in this study all pertain to the WATER domain, several water-related words are also regarded by other scholars as NATURAL FORCE, serving as an alternate source domain to illuminate the target domain. For instance, Kazemian and Hatamzadeh [44] assert that when we speak of the coronavirus as a destructive storm or flood, we perceive it as A NATURAL FORCE. Charteris-Black [51] also argues that words such as *tsunami*, *surge*, *tide*, *overwhelm*, and others, which involve “the movement of vast amounts of water” [51], not only belong to the WATER domain but also fall within a broader domain of NATURAL FORCE. This domain encompasses not only water-related words but also words that signify the experience of nature’s force, such as *overwhelm*, *batter*, and *strike*. The reason these words can be considered as NATURAL FORCE is that they all convey the notion of one entity exerting force upon another. Thus, from this perspective, the source domain for the water-related metaphors employed in the present study is multifaceted. When these words are framed within the context of the force of nature, the coronavirus is construed as being caused by non-human, “objective” forces that demand urgent responses from governments and societies.

Earlier studies have covered the topic of what makes a metaphor effective from several angles, demonstrating that metaphors are useful when (1) a prominent knowledge structure is brought to mind by the source domain (or feeling); (2) speaking members of a certain linguistic group are familiar with this information; and (3) it is reasonable in a certain culture to compare the target and source domains [52–59]. Each of these aspects is seen in the WATER metaphor. WATER evokes in our mind a salient knowledge structure.

The purpose of viruses is not to harm or eliminate their hosts. A virus’s goal is life, not death. Since viruses require living cells to multiply over time, they have developed transmission techniques that make it simple and effective to locate living hosts. Ideally, a pathogenic virus should infiltrate a live system and have enough time to replicate numerous times before being destroyed by the host’s immune system. If the host lives, the virus will too. The issue with pathogenic viruses, especially those that the host has never encountered before, is that the body’s state can occasionally be altered beyond the point at which the body can continue to function (especially weakened bodies). Therefore, the body’s response to the virus, rather than the virus itself, causes death. Walker [60] also contends that bacteria have shaped life on Earth for thousands of years and are ancient. As a result of our crucial microbiomes, dietary habits, and habitats, humans also exist biologically and socially inside the microbial world.

Humans and viruses are intertwined, and the connection between humans and the virus can be described as intra-action, according to Barad’s [61] terminology. To understand this profound interdependence between entities, American physicist and feminist philosopher of science, Barad [53], introduced the notion of intra-action. Her agential realism approach heavily depends on the concept of “intra-action”, which pertains to the mutual composition of interrelated agencies.

In contrast to the conventional notion of “interaction”, which assumes that different individual agencies must first come into contact, the concept of intra-action acknowledges that distinct agencies do not come into contact before they emerge through their intra-action. It is essential to note that the “different” agencies are only different in relation to one another, not in a strict sense. In other words, agencies do not appear as distinct parts; rather, they are only distinguishable in reference to their mutual interaction. According to this perspective, humans and viruses are not independent, autonomous entities that exist before their interaction, but rather, they are distinct only in a relational sense. In the PANDEMIC IS WAR metaphors, the warriors and the enemy are separate in an absolute sense, whereas in the PANDEMIC IS WATER metaphor, people and WATER (viruses) are only distinct concerning their mutual entanglement. Human beings and WATER (viruses) do not exist as individual elements but are entangled.

As long as human beings strive to eliminate and overcome the virus, we cannot break free from the SDM cognitive model. In the WAR metaphor, we attempt to defeat the enemy. Only by acknowledging that humans and viruses are not opponents but are entangled, can we abandon the SDM cognitive model. If we use the metaphor of the virus as WATER, the sharp differentiation between them dissolves.

Most importantly, since the PANDEMIC IS WATER metaphor is based on WDM, there is less confrontation between humans and the pandemic in areas where floods and tsunamis are not common. Therefore, WATER metaphors eliminate the dualism that exists in WAR metaphors. In the WAR frame, the pandemic/virus is metaphorically depicted as an “enemy” that needs to be defeated. This oversimplification is based on SDM, as illustrated at the outset. In contrast, the WATER frame provides a more vivid and effective way to mobilize people to prevent pandemic transmission. For example, quarantine is a necessary measure to control the pandemic transmission but may cause mental health issues such as stress, anxiety, feelings of depression, insomnia, and anxiety. If expressed in the WATER frame, as in Example 14, those mental problems may be alleviated by considering quarantine as a “life *raft*”.

From the arguments mentioned above, we can conclude that the WATER metaphor is suitable and practical for discussing the pandemic except in regions where floods and tsunamis are common. The PANDEMIC IS WATER metaphor promotes a new mindset that is open to all, compassionate, helpful, supportive, progressive, and unites individuals. It has the potential to enable new ways of behaving and participating in society.

## Conclusions

As stated at the beginning of this paper, there has been limited focus on the functions of WATER metaphors. In order to address this research gap, the present study examined the CC as a case study, utilising Pragglejaz Group’s MIP [48] and Wang’s WDM [32, 35].The study concludes that WATER metaphors can be flexibly employed for multiple purposes, mainly to distinguish between various pandemic periods, explain the occurrence and performance of the pandemic, outline the impact of COVID-19 on life, mobilise the public to take measures for reducing contagion, and convey danger. This study highlights the multiple functions that WATER metaphors played during the COVID-19 pandemic in communicating complex information and influencing public opinion. In areas where floods and tsunamis are common and cause significant property damage and fatalities, the applicability of the WATER metaphor may differ. Depending on the specific cultural, social, and contextual aspects at play, the perception of WATER metaphors in such areas may be positive or bad. We acknowledge the necessity for further studies that take into account the unique cultural, contextual, and socioeconomic characteristics of those places in order to fully address the suitability of WATER metaphors in regions vulnerable to water-related disasters. By doing so, it would be possible to have a deeper understanding of how WATER metaphors are interpreted and applied in these situations.
